# Learning to synchronize: How biological agents can couple neural task modules for dealing with the stability-plasticity dilemma

**DOI:** 10.1371/journal.pcbi.1006604

**Published:** 2019-08-20

**Authors:** Pieter Verbeke, Tom Verguts

**Affiliations:** Department of Experimental Psychology, Ghent University, Ghent, Belgium; Harvard University, UNITED STATES

## Abstract

We provide a novel computational framework on how biological and artificial agents can learn to flexibly couple and decouple neural task modules for cognitive processing. In this way, they can address the stability-plasticity dilemma. For this purpose, we combine two prominent computational neuroscience principles, namely Binding by Synchrony and Reinforcement Learning. The model learns to synchronize task-relevant modules, while also learning to desynchronize currently task-irrelevant modules. As a result, old (but currently task-irrelevant) information is protected from overwriting (stability) while new information can be learned quickly in currently task-relevant modules (plasticity). We combine learning to synchronize with task modules that learn via one of several classical learning algorithms (Rescorla-Wagner, backpropagation, Boltzmann machines). The resulting combined model is tested on a reversal learning paradigm where it must learn to switch between three different task rules. We demonstrate that our combined model has significant computational advantages over the original network without synchrony, in terms of both stability and plasticity. Importantly, the resulting models’ processing dynamics are also consistent with empirical data and provide empirically testable hypotheses for future MEG/EEG studies.

## Author summary

Artificial and biological agents alike face a critical trade-off between being sufficiently adaptive to acquiring novel information (plasticity) and retaining older information (stability); this is known as the stability-plasticity dilemma. Previous work on this dilemma has focused either on computationally efficient solutions for artificial agents or on biologically plausible frameworks for biological agents. What is lacking is a solution that is both computationally efficient and empirically testable on biological agents. Therefore, the current work proposes a computational framework on the stability-plasticity dilemma that provides empirically testable hypotheses on both neural and behavioral levels. In this framework, neural task modules can be flexibly coupled and decoupled depending on the task at hand. Testing this framework will allow us to gain more insight in how biological agents deal with the stability-plasticity dilemma.

## Introduction

Humans and other primates are remarkably flexible in adapting to constantly changing environments. Additionally, they excel at integrating information in the long run to detect regularities in the environment and generalize across contexts. In contrast, artificial neural networks (ANN), despite being used as models of the primate brain, experience significant problems in these respects. In ANNs, extracting regularities requires slow, distributed learning, which does not allow strong flexibility. Moreover, fast sequential learning of different tasks typically leads to (catastrophic) forgetting of previous information (for an overview see [1]). Thus, ANNs are typically unable to find a trade-off between being sufficiently adaptive to novel information (plasticity) and retaining older information (stability), a problem known as the stability-plasticity dilemma.

In recent years, a wide variety of solutions have been provided for this stability-plasticity dilemma. These solutions can broadly be divided in two classes. The first class is based on the fact that catastrophic forgetting does not occur when tasks are intermixed. Thus, one solution is to keep on mixing old and new information [2–5]. [3] suggested that new information is temporarily retained in hippocampus. During sleep (and other offline periods), this information is gradually intermixed with old information stored in cortex. This framework inspired subsequent computational and empirical work on cortical-hippocampal interactions [6–8].

The second class of solutions is based on the protection of old information from being overwritten. Protection can occur, first, at the level of synapses. For example, [9] combined a slow and fast learning system, with slow and fast weights reflecting long- and short-time-scale contingencies, respectively. This allows the network to both extract stable regularities (slow learning system) and flexibly adapt to fast changes in the environment (fast learning system). Another recent idea is to let synapses (meta-)learn their own importance for a certain task [10], [11]. Weights that are very important for some task are not allowed to (and thus protected from) change. Hence, information encoded in those weights is preserved. Second, protection can also be implemented at the level of (neural) activation. The most straightforward approach to implement such protection is to orthogonalize input patterns for the relevant tasks [12], [13]. Another approach to achieve protection at the level of neural activation, is gating. This means that only a selected number of network nodes can be activated. Because weight change depends on co-activation of relevant neurons [14], [15], this approach protects the weights from changing. For example, [16] proposes that in each of several tasks a (randomly selected) 80% of nodes is gated out, thus effectively orthogonalizing different contexts. They showed that synaptic gating allowed a multi-layer network to deal with several computationally demanding tasks without catastrophic forgetting.

Crucially, it remains unknown how biological agents deal with this dilemma. The current study aims to provide a novel computational framework focused on biological agents that makes empirically testable predictions at MEG/EEG level. For this purpose, we combine two prominent principles of computational neuroscience, namely Binding by Synchrony [17–20]) and Reinforcement Learning (RL; [21], [22]). In BBS, neurons are flexibly bound together by synchronizing them via oscillations. This implements selective gating (e.g., [23]) in which synchronization enhances the communication between neuronal groups (gates are opened) and desynchronization disrupts the communication between neural groups (gates are closed). In sum, BBS allows the model to flexibly alter communication efficiency on a fast time scale. By using RL principles, the model can learn autonomously when neurons need to be (de)synchronized.

In the modeling framework, BBS binds relevant neural groups, called (neural task) modules, and unbinds irrelevant modules. This causes both efficient processing and learning between synchronized modules; and inefficient processing and absence of learning between desynchronized modules. The resulting model deals with the stability-plasticity dilemma by flexibly switching between task-relevant modules and by retaining information in task-irrelevant modules. An RL unit [24] uses reward prediction errors to evaluate whether the model is synchronizing the correct task modules.

In order to test the generalizability of our framework, we apply it to networks containing modules that learn via three classic synaptic learning algorithms, namely Rescorla-Wagner (RW; [15], [25]), backpropagation (BP; [26]) and Restricted Boltzmann machines (RBM; [27]). The RW algorithm [25] is one of the most well-known and basic supervised-learning algorithms in cognitive neuroscience. Here, on each trial, an error term is computed based on the discrepancy between a model-generated output pattern and some target output pattern. Learning consists of using this error term for finding a weight configuration that minimizes the average error across trials. This algorithm is typically fast and efficient for learning simple (i.e., linearly separable) input-output associations. Hence, it has no problems with plasticity. However, while learning one set of input-output associations (set B), the algorithm may unlearn another, currently irrelevant set of input-output associations (set A). Thus, when set A becomes relevant again, it will have to relearn it. In sum, the RW algorithm does suffer from a lack of stability, but due to its high plasticity it might have only minor problems with respect to the stability-plasticity dilemma, especially when the learning rate is high. In this case, it might relearn the forgotten information (set A) so fast that also the stability problem is negligible. Nevertheless, the RW algorithm suffers from some severe limitations on the complexity of problems that it can solve. It is very efficient in dealing with linearly separable input-output associations, but cannot deal with more complex, not linearly separable, problems.

This limitation of the RW algorithm is solved in BP [26]. Similar to RW, learning with BP consists of using the error term for finding a weight configuration that minimizes the average error across trials. Relative to RW, this algorithm is able to solve a much wider range of problems. In particular, it can also solve nonlinearly separable problems. It does this by adding hidden layers between input and output. For training the weights toward the hidden layers, BP propagates the error term backwards from output toward the hidden (i.e., deeper) layers in the network. Crucially, sequential learning of input-output associations poses severe computational problems on the BP algorithm. Because the number of (interdependent) weights that should be adjusted to solve a problem is much higher, the algorithm learns much slower. Hence, if the learning rate is low, new learning can be very slow, causing a lack of plasticity. If the learning rate is very fast, on the other hand, this problem is mitigated but there is no stability in the model. This is because, similar to RW, the learning algorithm will adapt all available weights and therefore overwrite previous information.

An algorithm that can also learn with hidden layers (and thus solve more complex problems) is RBM. Despite the algorithmic differences, RBM suffers from the same stability-plasticity dilemma as BP. To further illustrate the generality of our framework, S1 Text show that our framework can also be applied to networks with modules that learn via RBM. For brevity, the main text restricts attention to RW and BP.

**Fig 1:**
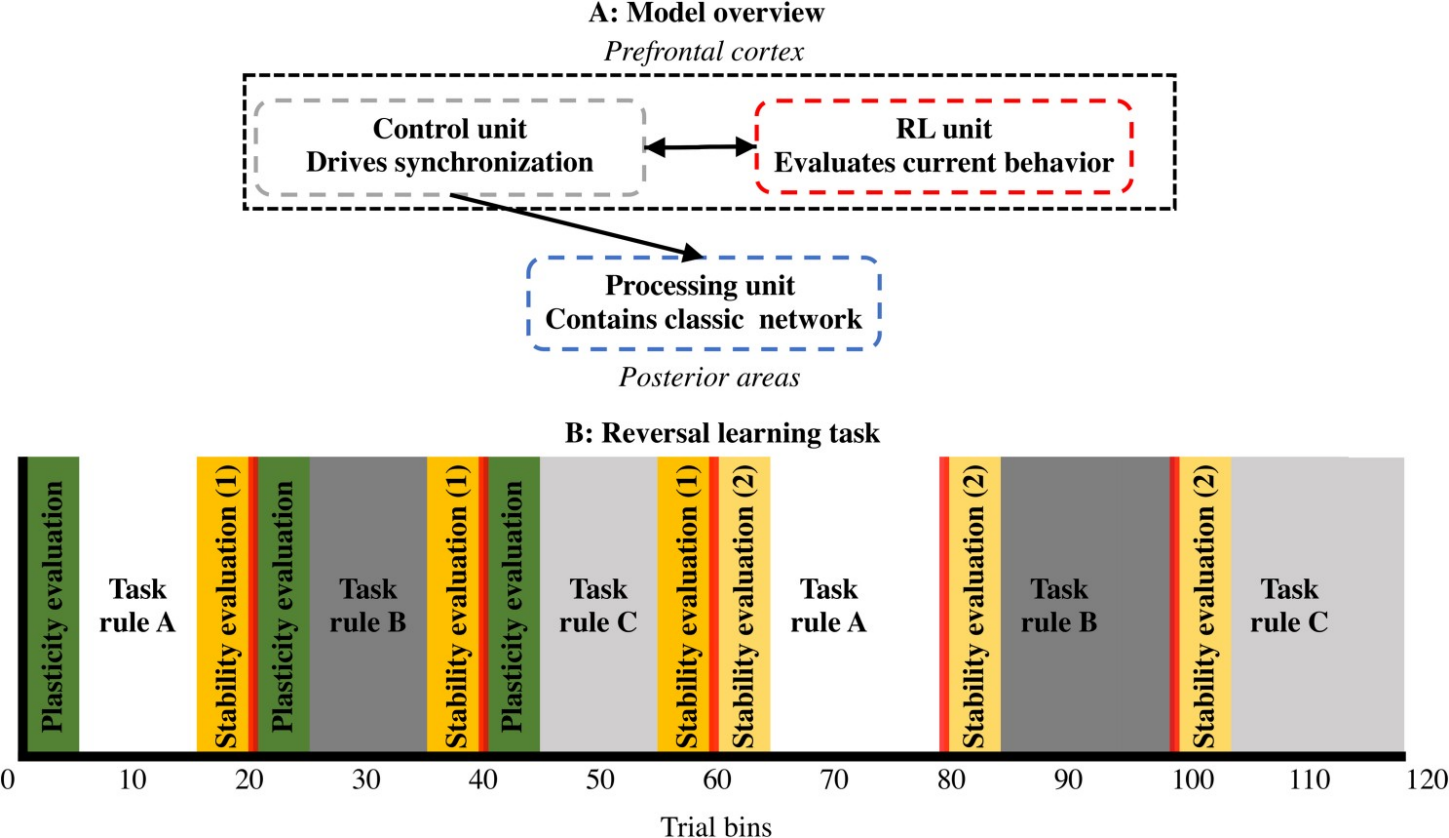
Model and task overview. **A:** General model overview. The model consists of 3 units. A Processing unit contains a classic neural network that learns the (reversal learning) task. The Control and RL units constitute a hierarchically higher network. Putative brain areas are shown in italic font. The Control unit drives synchronization of oscillations in the Processing unit. The RL unit evaluates current behavior in order to signal to the Control unit what should be synchronized in the Processing unit. **B:** Reversal learning task. The task alternates between 3 task rules (A, B, C) across 6 task blocks with task sequence ABCABC. Plasticity is measured during the first 5 trial bins of the first task block in which a task rule is presented (green bars). Stability is measured as the difference between the last 5 trial bins of the first task block in which a task rule is presented, and the first 5 trial bins of the second task block in which a task rule is presented.

**Fig 2:**
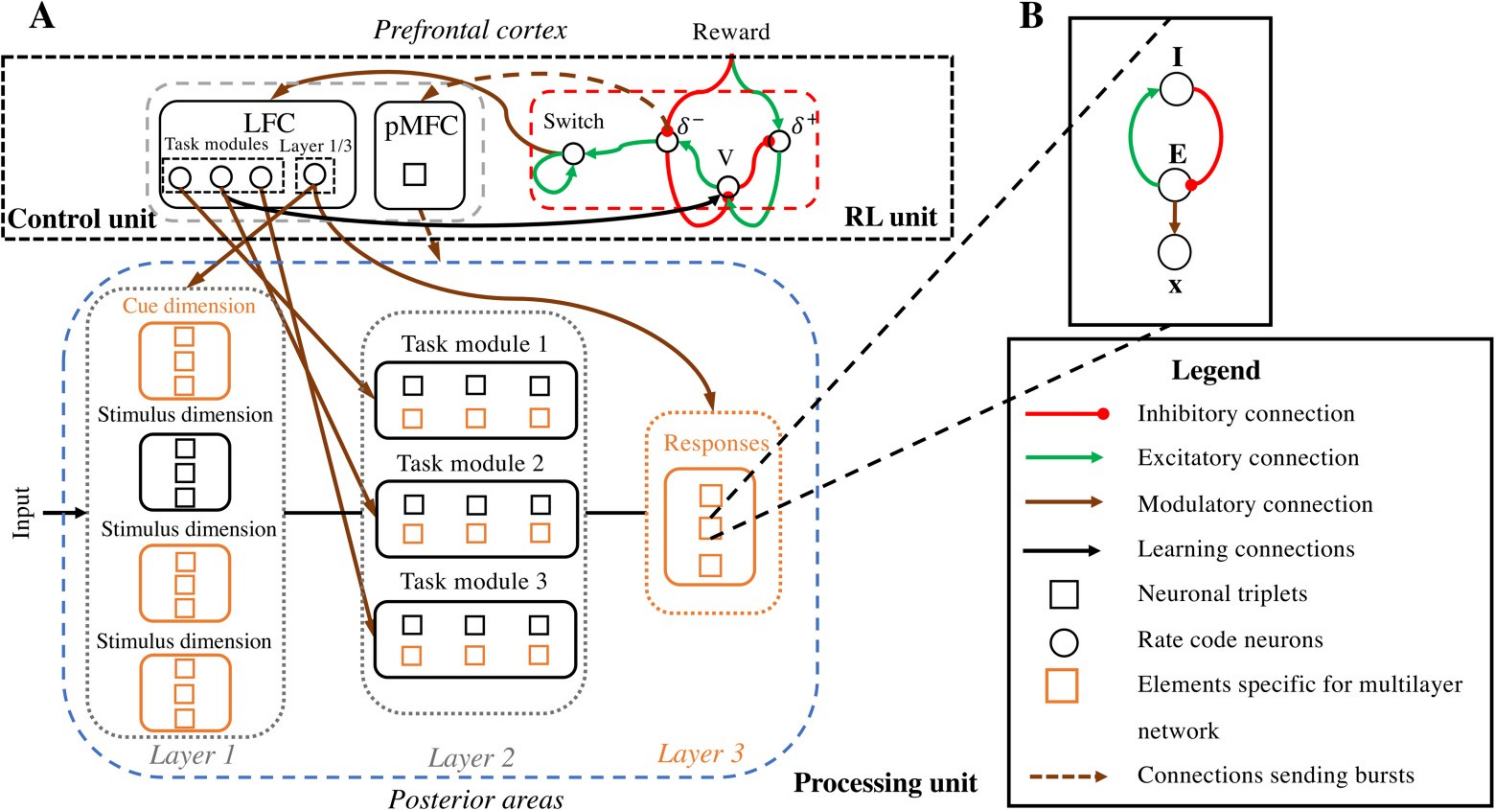
Detailed overview of the model. **A:** The model. A detailed version of the model in Fig 1A is shown. The model consists of 3 units. A Processing unit is localized in posterior processing areas and contains a classic neural network. This network contains 3 layers (of nodes) for the BP model and 2 layers for the RW model. Layer 1 contains nodes that are activated by external input. At layer 2, modularity is implemented. This layer is divided in 3 task modules, one for each task the model has to execute. In the BP model, the nodes in these task modules represent hidden nodes; for the RW model these nodes represent response options. Layer 3 only occurs in the BP model and contains three response options. The Control unit consists of two parts. Here, the LFC contains 4 task neurons; 3 neurons point to a specific task module in the Processing unit that should be synchronized or desynchronized. A fourth neuron points to layer 1 and 3, to indicate that task modules should be (de)synchronized with these layers. The pMFC of the Control unit contains one single node that sends bursts in order to (de)synchronize modules in the Processing unit in line with the pointers sent by the LFC. The RL unit contains four neurons. One neuron (*V*) learns to assign a value to the task modules. Two other neurons (*δ—*, *δ* ^***+***^) compare this value to external reward, in order to compute prediction errors. Negative prediction errors are accumulated in the Switch neuron in order to make a stay/switch decision, which it signals to the LFC. Additionally, the negative prediction error neuron signals to the pMFC (by giving bursts) that it should increase control. **B:** Neuronal triplet. Every square node in A consists of a triplet of neurons. Each such node consists of a phase-code pair (*E*, *I*) which, because of its excitatory (*E*)—inhibitory (*I*) coupling, oscillates at a certain frequency. These oscillations modulate the excitability of their rate code neuron (*x*) in line with the BBS hypothesis.

The full model consists of three units (Fig 1A). The Processing unit contains a network consisting of a number of task-specific modules; the two learning algorithms (RW or BP) are implemented between modules of the Processing unit. In addition, RL and Control units together form an hierarchically higher network modeled after basal ganglia/primate prefrontal cortex [28]. The RL unit (modeling ventral striatum/ anterior medial frontal cortex (aMFC)) evaluates behavior. More specifically, it learns to assign a value to a specific task module (how much reward it receives by using this module) and compares this value with the externally received reward to compute prediction errors. Additionally, the RL unit has a Switch neuron (see Fig 2A). This Switch neuron computes a weighted sum of negative prediction errors across trials. When this sum reaches a threshold of .5, it signals the need for a strategy switch to the Control unit (see Methods for details). This Control unit drives neural synchronization in the Processing unit. One part of the Control unit (modeling lateral frontal cortex (LFC)) contains task neurons that point to task modules in the Processing unit [29]; another part (modeling posterior medial frontal cortex (pMFC)) synchronizes task modules based on those task neurons [30]. Crucially, LFC and pMFC both use prediction error information, but on different time scales. The pMFC uses prediction errors on a fast time scale to enhance control over the synchronization process as soon as a negative prediction error occurs. In contrast, the LFC uses prediction errors on a slow time scale to know when the task rule has changed and a switch of modules is needed.

In order to drive neural synchronization between task modules in the Processing unit, we rely on the idea of binding by random bursts [30–32]. Here, applying positively correlated noise to two oscillating signals reduces their phase difference. In addition to implementing binding by random bursts, the current work also implements unbinding by random bursts. In particular, applying negatively correlated bursts increases the phase difference between oscillating signals and thus unbinds (i.e., dephases) the two signals.

We test our model on a (cognitive control) reversal learning task. Here, each hierarchically lower algorithm (RW or BP; in the Processing unit) sequentially learns different task rules. The relevant task rule changes across task blocks (Fig 1B). The model must detect when task rules have changed, and flexibly switch between different rules without forgetting what has been learned before. We divide the task in six equally long task blocks. In the first three blocks, the model should learn three different new task rules (rule A, B and C in blocks 1, 2 and 3 respectively). In the second half, the model has to switch back to the previously learned rules (rule A, B and C in blocks 4, 5 and 6 respectively; see also Fig 1B).

For the RW network, we use a one-dimensional task. Here, on each trial one out of three stimulus features is activated. For every task rule we link a stimulus feature to a response option. More specifically, in task rule A, feature 1 (F1) is associated to response 1 (R1), feature 2 (F2) to response 2 (R2) and feature 3 (F3) response 3 (R3). In task rule B, F1 is associated to R2, F2 to R3 and F3 to R1. Task rule C associates F1 to R3, F2 to R1 and F3 to R2. For the BP network, a multi-dimensional task is used. Here, on each trial multiple stimulus features are activated. More specifically, the task utilizes four dimensions. Every dimension has three features. One of the dimensions represents a cue that indicates which out of the other three (stimulus) dimensions is relevant on the current trial. In line with the one-dimensional task, the 3 stimulus features of each dimension are within each task rule linked to one response option.

The one-dimensional task (for RW) consists of 360 trials; the multi-dimensional task (for BP) consisted of 3600 trials. For comparison, we divided each task sequence in 120 trial bins for analysis and plotting. Fig 2A illustrates the detailed model for both tasks. We compare our combined (henceforth, full) models with models that use no synchronization (i.e., only contain the Processing unit; called no-synchrony models). We evaluate plasticity as the ability to learn a new task; and stability as the interference from learning a new task toward performance on the old task (see Fig 1B and Methods).

## Results

### Model architecture

#### Overview

An overview of the model is given in Fig 2A. In line with Fig 1A, a Processing unit, a Control unit and an RL unit are shown. The Processing unit contains a classic network with 2 layers (RW) or 3 layers (BP). At the second layer, each module groups all nodes that are relevant for one task rule.

The Control unit consists of the LFC and pMFC. The LFC holds pointers that indicate which modules should be (de)synchronized. This synchronization process is then executed by the binding by random burst principle [30–32]. In the model, a theta-frequency-paced signal produced in the pMFC is responsible for sending these bursts.

The RL unit (adopted from an earlier RL model [24]) computes an expected reward (*value*, *V*) for the currently used task module. This value is then compared to an external *Reward* signal in order to compute prediction errors (*δ* ^***-***^, *δ* ^*+*^). The negative prediction error signal is then propagated to both the Switch neuron and to the pMFC. A single negative prediction error increases the control signal in the pMFC (see Eq (8)). Instead, the Switch neuron evaluates the prediction error signal on a slower time scale (see Eq (12)). When activation in the Switch neuron reaches a threshold, it signals the need for a switch to the LFC in the Control unit, and resets its own activation to zero. Correspondingly, the LFC will change the signal to the Processing unit, and synchronize another task module (see Methods). We elaborate on this Switch neuron in the Model dynamics and performance section.

#### Neuronal triplets

In the Processing unit and the pMFC part of the Control unit, all processing happens in oscillatory nodes. As presented in Fig 2B, each oscillatory node contains one neuronal triplet. Each triplet contains one classical rate code neuron (with activation *x*_*i*_) which receives, processes and transmits information; and one pair of phase code neurons (*E*_*i*_, *I*_*i*_) which organizes processing in the rate code neurons. In line with previous work [30], excitatory neurons are updated by

$${\Delta E}_{i}(t)=-C\times I_{i}(t)-Damp\times J(r>r_{min}){\times E}_{i}(t)+B_{i}(t)$$ (1)

where Δ*E*(*t*) = *E*(*t* + Δ*t*)–*E*(*t*); and inhibitory neurons are updated by

$${\Delta I}_{i}(t)=+C\times E_{i}(t)-Damp\times J(r>r_{min}){\times I}_{i}(t)$$ (2)

The E and I neurons are thus coupled by a parameter *C*, causing them to oscillate. The strength of the coupling (*C*) determines the frequency of the oscillations, *C*/(2π) [30], [33]. Task-relevant modules in the Processing unit must be bound together. Previous research has proposed that such binding is supported by oscillations in the gamma-frequency band (30–70 Hz; [19]). We therefore chose a value for *C* corresponding to a frequency of ~40 Hz. In the pMFC, which executes top-down control, the value of *C* is such that oscillations are at a 5Hz (theta-) frequency, in line with suggestions of previous empirical work [34], [35]. The variable *t* refers to time, and Δt refers to a time step of 2 msec. The radius (*r*^2^ = *E*^2^*+I*^2^) of the oscillations in the Processing unit are attracted towards the value *r*_min_ = 1. This is implemented by the terms *Damp*×*J*(*r>r*_min_)×*E*_*i*_(*t*) in Eq (1) and *Damp*×*J*(*r>r*_min_)×*I*_*i*_(*t*) in Eq (2). Here, *J*(.) is an indicator function, returning 1 when the radius is higher than the value of *r*_min_, and 0 otherwise. The damping parameter, *Damp* = .3, determines the strength of attraction. Since a constant high pMFC power is computationally suboptimal and empirically implausible [36], the radius of the pMFC was attracted towards a smaller radius, *r*_min_ = .05. The damping parameter was set to *Damp* = .003, in order to let the amplitude of the pMFC oscillations decay slowly across trials. We elaborate on these parameters in the Results and Discussion section.

Excitatory neurons additionally receive a burst *B*_*i*_(*t*). For the nodes in the Processing unit, these bursts were determined by a combination of the LFC and pMFC signal, multiplied with standardized-Gaussian noise (*U*):

$$B_{i}(t)={LFC}_{i}\times pMFC(t)\times U(t)$$ (3)

**Fig 3:**
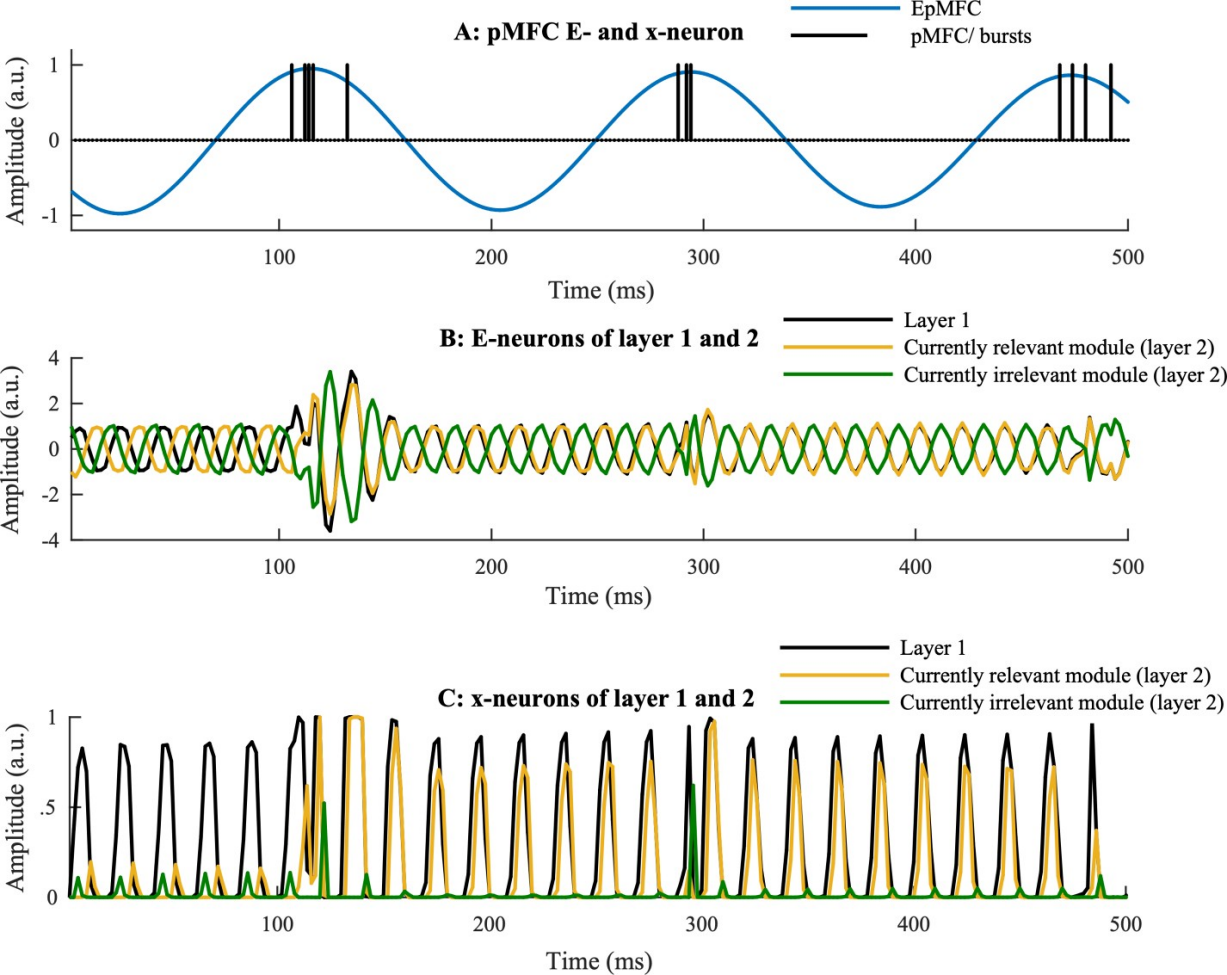
Neuronal triplets. **A:** The pMFC. In the pMFC, the phase code neurons oscillate at a 5 Hz frequency. The rate code neuron of the pMFC gives bursts to the Processing unit. Every time the E-neuron reaches a high amplitude, the probability of a burst becomes high. **B:** E-neurons of the Processing unit. In the Processing unit, the phase code neurons oscillate at a faster gamma-frequency. It is illustrated how a burst leads to (de)synchronization of oscillations that at first were not (de)synchronized. **C:** Rate code neurons in the Processing unit. Consequences of synchronization between the phase code neurons can be observed in the rate code neurons. At first, only the neuron of layer 1 is activated because it receives a constant external input signal. Importantly, this activation is modulated by *G*(*Ei*) in Eq (4). As a consequence, as long as the E-neurons are not synchronized, communication between the corresponding rate code neurons is very inefficient; but when the E-neurons are synchronized, communication between the corresponding rate code neurons is efficient.

As can be observed in Fig 3B, these bursts lead to synchronization between modules (and hence nodes) that are relevant (which receive the same LFC signal); and desynchronization between modules (and hence nodes) that are irrelevant (which receive opposite LFC signals). Note that bursts also introduce noise, hence it is optimal to limit the number of bursts. This is one of the reasons why the amplitude of the pMFC decays slowly over trials.

The rate code neurons in the Processing unit are updated by

$$\Delta x_{i}(t)=-x_{i}(t)+f({net}_{i}-bias)\times G(E_{i}(t))$$ (4)

The term -*x*_*i*_(*t*) will cause fast decay of activation in absence of input. According to this equation, the activation of the rate code neuron at every time step is a function of the net input (*net*_*i*_) for that neuron, multiplied by a function of the excitatory phase code neuron [30],

$$G(E_{i}(t))=\frac{1}{1+e^{\left(-5\times (E_{i}(t)-.6)\right)}}$$ (5)

How this function affects the rate code neurons (*x*) is illustrated in Fig 3C. In the BP network, the rate code neurons have a sigmoid activation function *f*(*net*_*i*_*-bias*) = $\frac{1}{1+e^{-({net}_{i}-bias)}}$. Additionally, these rate code neurons receive a *bias* = 5 to set activation to (approximately) zero in absence of input. In the RW network, the rate code neurons have no bias and follow a linear activation function; *f*(*net*_*i*_*—bias*) *= net*_*i*_.

As described in Eq (3), the synchronizing bursts that are sent to the Processing unit are a combination of an LFC and a pMFC signal. Here, LFC represents a pointer that takes on a value of 1 for the module that should be synchronized and -1 for modules that should be desynchronized. The pMFC-part of the equation corresponds to the rate code neuron of the pMFC triplet, which follows

pMFC(t)∼Be(p) (6)

This equation represents a Bernoulli process *Be*(*p*) which is 1 with probability *p*. The probability

$$p=\frac{1}{1+e^{\left(-10\times (E_{pMFC}(t)-1)\right)}}$$ (7)

is a sigmoid function which has its greatest value when the *E*_*pMFC*_ is near its top and its amplitude is sufficiently strong. Hence, every time the oscillation of the *E*_*pMFC*_-neuron reaches its top, the probability of a burst becomes high. Thus, bursts are phase-locked to the theta oscillation (see [30] for more details). An illustration of the processes in the neuronal triplets of both the pMFC and Processing unit is presented in Fig 3.

The pMFC not only sends burst but also receives bursts. Here, the burst signal received by the pMFC is determined by the negative prediction error signal of the previous trial,

$$B_{pMFC}(n,t)={\delta^{-}}_{n-1}*∼Be(e^{\frac{{-(t-200)}^{2}}{2\times 25^{2}}})$$ (8)

Here, the burst signal at one time point in one trial (*n*) is determined by the size of the negative prediction error at the previous (*n*—1) trial, by a Bernoulli process Be(*P*(*t*)) which is 1 with probability *P*(t) (and 0 otherwise). The probability *P*(*t*) is shaped like a Gaussian distribution that peaks at 200 ms with a standard deviation of 25 ms, representing a communication delay between the RL unit and the pMFC. This delay does not necessarily represent the time of a direct information transfer between the pMFC and *δ* ^-^, but is rather set to this value in order to connect with empirical data showing feedback-related EEG-activity at approximately 200 ms after feedback presentation [37–39]. Hence, when a trial elicited a negative prediction error, bursts are sent to the excitatory neuron of the pMFC. Consequently, these bursts have the size of the negative prediction error and are most likely to occur at 200 ms after feedback. This burst signal will increase the amplitude of the pMFC phase code neurons when a negative prediction occurs, after which it will decay towards *r*_*min*_.

### Model dynamics and performance

**Fig 4:**
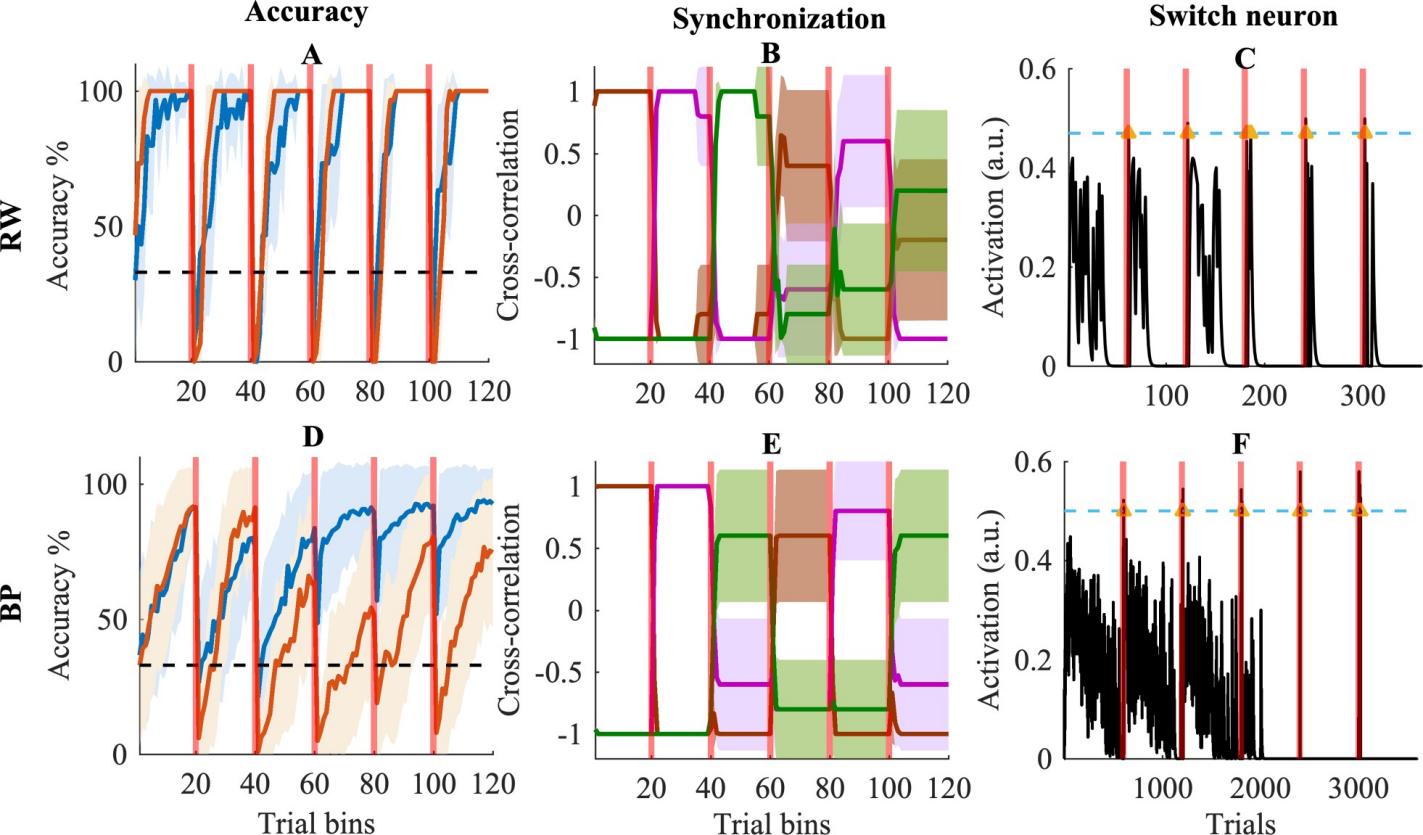
Model data. Model dynamics are shown for simulations with a learning rate of .2. In column 1 (panels A and D) binned accuracy is shown for the full (in blue) and no-synchrony (in orange) model. The horizontal dashed black line indicates accuracy at chance level. In column 2 (panels B and E), brown lines represent synchronization values for the initially (randomly) chosen task module, magenta lines for the module that was chosen secondly, and green lines for the third module. In column 3 (panels C and F), activity of the Switch neuron (see Fig 2A) is shown for one selected simulation of the model (in black). Blue horizontal dashed lines indicate the threshold of the Switch neuron and the yellow arrows mark data points above the threshold. In all panels, red vertical transparent lines indicate task switches and shades indicate 95% confidence intervals.

In Fig 4A, the accuracy evolution across all task blocks is plotted for both the full and no-synchrony RW model with a slow learning rate, β = .2, for the simple (linearly separable) task. The full model is marginally slower in learning new task rules. However, when the model needs to switch back to a previously learned rule (task blocks 4–6) we observe a minor advantage for the full model in the first trials, since it does not have to relearn the task.

A very different picture emerges for the complex (nonlinearly separable) task. Fig 4D shows the accuracy of the full and no-synchrony BP model. During the first task block, the no-synchrony and full model perform similarly. When the task rule switches for the first time (i.e., after the first task block), the drop in accuracy is slightly larger for the no-synchrony model than for the full model. This is caused by the fact that the no-synchrony model has to learn task rule B with weights that were pushed in a direction opposite to those that are optimal for task rule A. Instead, the full model switches to another task module and starts learning from a random weight space. A similar phenomenon occurs after the second rule switch.

For the following task switches, the model has to switch back to rules it already learned before; it is here that the full potential of the full model emerges. The full model can switch back to a previous module, where all old information was retained. Instead, the no-synchrony model has catastrophically forgotten the first task rule and must hence relearn it.

#### Synchronization of modules

Fig 4B represents the synchronization between the input layer and different task modules for the RW model. Here, we see that the model performs quite well in synchronizing task-relevant and in desynchronizing task-irrelevant modules. Additionally, the model is able to flexibly switch between modules at the correct point in time. As is illustrated by the broader confidence intervals, the model needs some time to switch back to a previously used module. Nevertheless, in general it does succeed in finding the correct task module. A similar pattern is observed in Fig 4E, where the synchronization of the BP model is shown.

#### The switch neuron

Fig 4C shows activation in the Switch neuron for the RW model. Here, two crucial observations can be made. First, when the model has to learn a task for the first time there is more activity in the Switch neuron. This reflects the learning process where many (negative prediction) errors occur. When the network has learned the task, activity in the Switch neuron decays towards zero because less errors are made. Hence, the fact that there is almost no activity in the Switch neuron when the model performs an already learned task, also demonstrates the stability of the model. Second, the Switch threshold of .5 is only reached during the first trials after a rule switch. This is because at this moment, many large prediction errors occur and accumulate. Once the Switch threshold is reached, activity in the Switch neuron decays towards zero. A similar process can be observed in Fig 4F where data for the BP model is shown.

#### Parameter exploration

**Fig 5:**
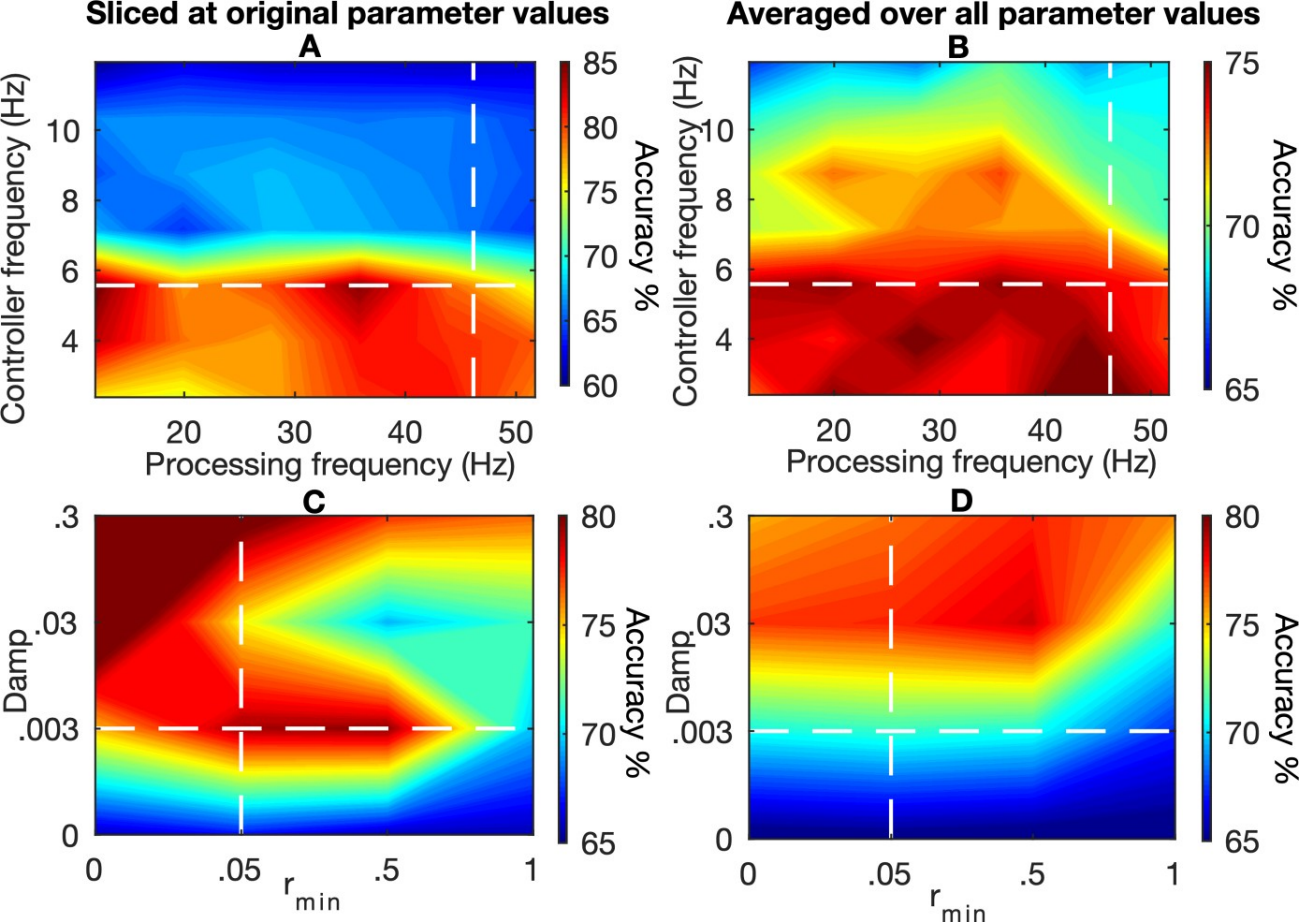
Parameter exploration. The first row (A-B) shows results for the combination of the Controller frequency and the Processing frequency. The second row (C-D) shows results for the combination of the *Damp* and *r*_*min*_ parameters. In the first column we show results where the other two parameters where kept constant at the original values that we used for other simulations (i.e., we slice parameter space in these two parameters). In the second row, results are shown where we average over all values used for the remaining parameters. Colors indicate mean accuracy over the whole task. The white dashed lines indicate the original parameter values.

Parameters in the current model were mainly set based on previous work [19], [30], [34–36]. We performed an additional simulation (see Methods for details) in which we explored the importance of the frequencies in the Processing unit and pMFC, and the decaying amplitude in pMFC. Results are shown in Fig 5. We observe that accuracy dramatically declines when the Controller frequency, determined by the *C* parameter in Eqs (1) and (2) for the pMFC, is too fast. Additionally, the model performs best when the *Damp* parameter is high and the *r*_*min*_ parameter is low (again, see Eqs (1) and (2)). Note that this does not mean that pMFC amplitude is always low because it still receives bursts (Eq (8)) when it makes errors. Nevertheless, performance is optimal when pMFC amplitude decays fast. The reason for this is the fact that bursts introduce noise to the system (see also Fig 3C). Therefore, the model performs better when the oscillation driving the bursts is slower and less strong, so that fewer bursts are given. We elaborate on these results in the Discussion.

### The stability-plasticity dilemma

**Fig 6:**
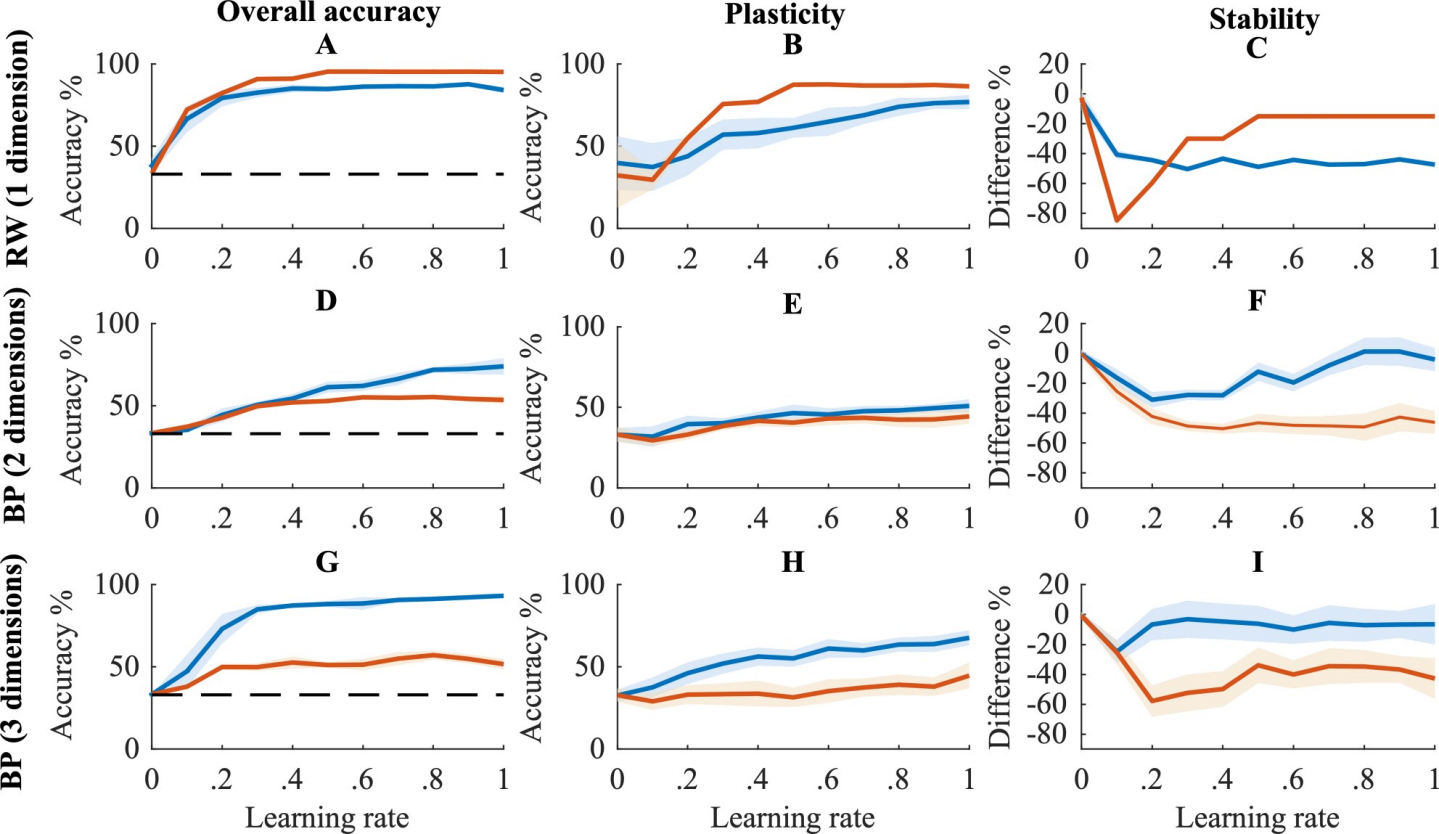
Performance of models on reversal learning task. Overall accuracy (A, D, G), plasticity (B, E, H) and stability (C, F, I) is shown across all learning rates for three tasks of increasing complexity (see Methods for details). Blue lines show means for the full model and orange lines represent the mean values for the no-synchrony models. The shades indicate the corresponding 95% confidence intervals. The horizontal black dashed line in A and D indicates chance level accuracy.

Fig 6 shows the overall accuracy, stability and plasticity of our full model and of the no-synchrony model for the two task structures discussed in the previous section (1- and 3-dimensional tasks). In order to gain more insight in how the model performance is affected by task complexity, we also show overall results for the BP model on a 2-dimensional task. Thus, we show results for tasks of increasing complexity, namely for 1 dimension (RW model), 2 dimensions (BP model) and 3 dimensions (BP model). Results of the RBM model are discussed in S1 Text.

#### RW

Fig 6A–6C shows similar overall accuracy for the full and no-synchrony RW models. When synaptic learning rates are slow (β = .1-.3), the full model has a better stability than the no-synchrony model. However, this advantage disappears for higher learning rates and the no-synchrony model shows a higher plasticity than the full RW model. In sum, when the task is very easy and the learning rate is very high, synchronization is not required.

#### BP

Fig 6D–6I show a clear advantage for the full relative to the no-synchrony BP model in overall accuracy as well as plasticity and stability. This advantage was present across all learning rates and for both tasks (i.e., with 2 and 3 dimensions). This advantage appears because the synchronization supports modularity, thus protecting information from being overwritten.

### Connecting to empirical data

As a model of how the brain controls its own processing, we next aimed at describing the relation between our model and previous empirical data, and provide testable hypotheses for future empirical work.

#### Theta power

**Fig 7:**
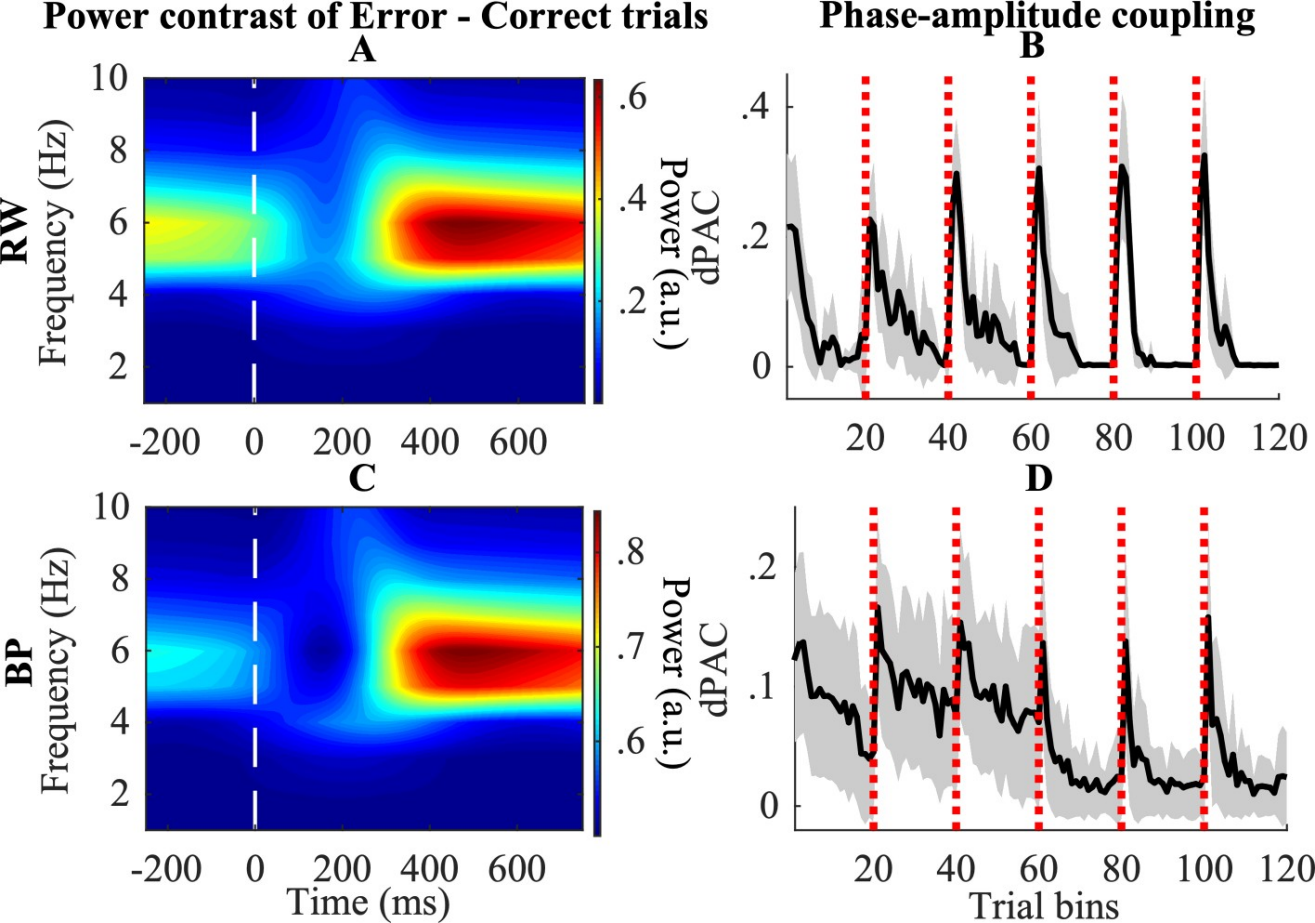
Connecting to empirical data. A, C: Contrast of error–correct trials is shown for post-feedback pMFC power in time-frequency spectrum. B, D: phase-amplitude coupling between pMFC theta-phase and gamma-amplitude in the Processing unit is shown. White vertical dashed lines indicate the moment of reward feedback. Red vertical transparent lines indicate task switches. Shades illustrate 95% confidence intervals.

As described before, theta power in the pMFC gradually decayed during the task. However, when a negative prediction error occurred, the pMFC network node received a burst (from *δ* ^*-*^; see Eq (8)), which increased pMFC amplitude again. In order to illustrate this process, we performed time-frequency decomposition of the signal produced by the pMFC node. More specifically, we were interested in theta power after feedback. We computed the contrast of power in the inter-trial interval after error and after correct trials in the time-frequency domain (see Methods for details). In accordance with previous empirical work (e.g., [35], [37], [39]), we observe increasing theta power, starting 200 ms after negative feedback, both for the RW (Fig 7A) and BP (Fig 7C) model.

#### Phase-amplitude coupling

Fig 7B and 7D illustrate the coupling between the phase of theta oscillations in the pMFC and gamma amplitude in the Processing unit. Again consistent with empirical data [34], [40], these plots show a clear increase in phase-amplitude coupling after a task rule switch. This is mainly caused by the fact that there are many negative prediction errors in these trials. These prediction errors increase theta power in the pMFC, which in turn increases the number of bursts received by the gamma oscillations in the Processing unit. This combination of events results in an increase of theta-gamma phase-amplitude coupling (PAC). Once performance of the model improves, less (negative prediction) errors occur. Hence, theta power slowly decreases, which decreases bursts to the processing unit and thus also PAC.

## Discussion

We described a computationally efficient and empirically testable framework on how biological and artificial agents may deal with the stability-plasticity dilemma. We combined two neurocomputational frameworks, BBS [17–19] and RL [21]. BBS flexibly (un)binds (ir)relevant neural modules; RL autonomously discovers when modules need to be (un)bound. Thus, the model could flexibly switch between different tasks (plasticity) without catastrophically forgetting older information (stability). We demonstrated that the model was consistent with several behavioral and electrophysiological (e.g., MEG/EEG) data. In the remainder, we first summarize the main model components, and point to plausible neural origins of each. Second, we discuss specific empirical predictions that are made by the model. Third, we discuss limitations and possible extensions. As a fourth and last point, we describe how the current work relates to previous computational modelling work.

**Fig 8:**
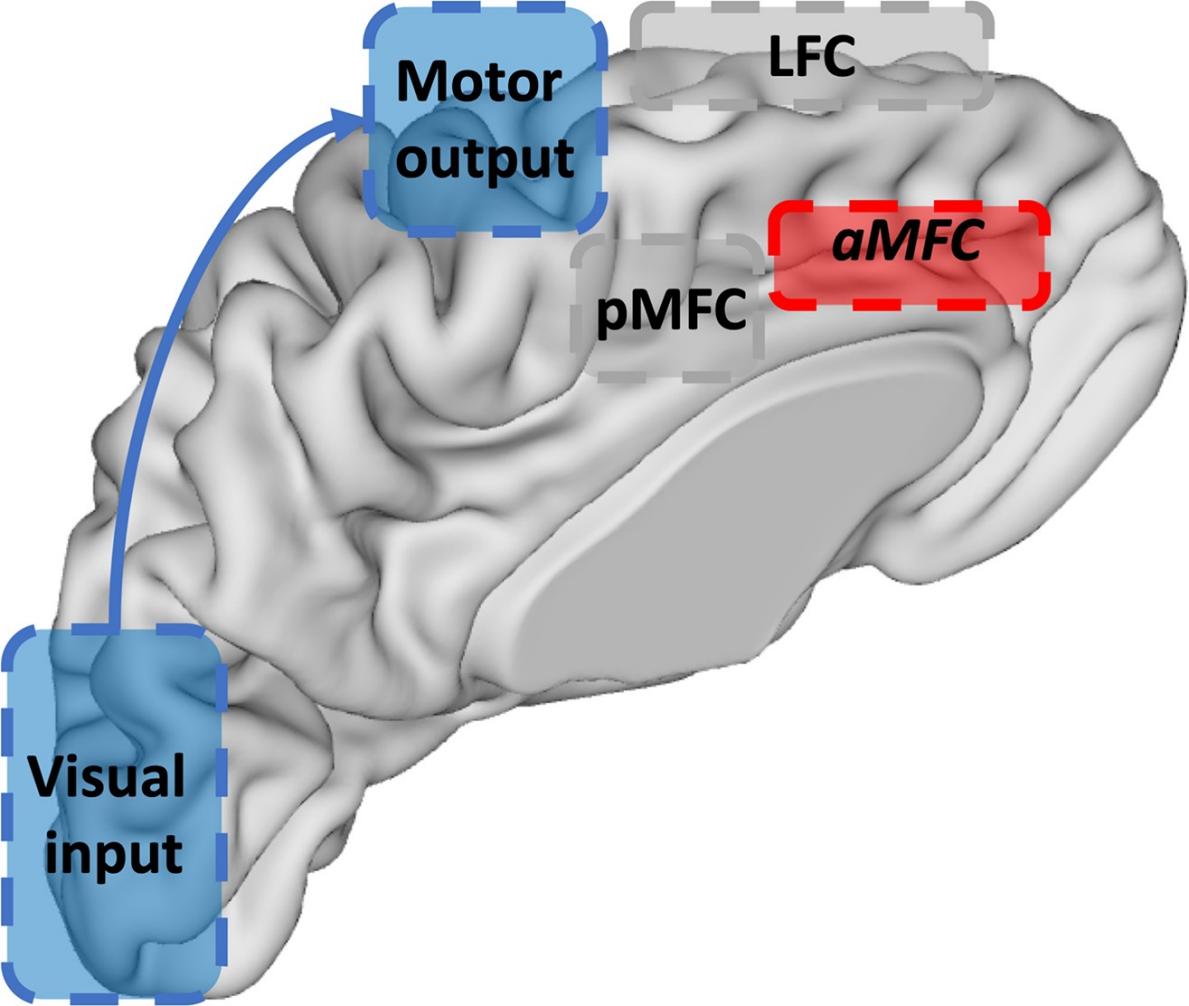
Suggestion of neural origins of three model units. The Processing unit (in blue) is situated at posterior cortical sites. In the case of a task in which stimuli are visually presented, and responses are hand movements, the Processing unit would consist of visual cortex and pre-motor (and intermediate) areas. The RL unit (in red) could be localized in aMFC (in combination with brainstem and frontopolar cortex (not depicted). The Control unit (in grey) consists of LFC and pMFC.

Plausible neural origins for all three model units are summarized in Fig 8. The Processing unit contains a task-processing network, trained by a classical learning rule (RW, BP, or RBM). Anatomically, its nodes can be localized in several posterior (neo-)cortical processing areas, depending on the task at hand (e.g., fusiform face area in a face-processing task). Its activity is strongly stimulus-dependent and synaptic strengths change slowly. The RL unit learns to attach value to specific task modules, based on prediction errors. Previous work with fMRI [24], [41] already used a probabilistic reversal learning paradigm to localize the brain areas involved in such value learning. This work localized the RL unit in MFC, which (with brainstem and striatum) is generally considered as an RL circuit [24], [42], [43]. Importantly, computations in this unit are not used for driving task-related actions, but for driving hierarchically-higher actions, namely to (de)synchronize task modules. This is in line with recent considerations of MFC as a meta-learner [44–47]. We tentatively call this unit aMFC, given this region’s prominent anatomical connectivity to autonomous regions [48]. There was also a Switch neuron in our model. Previous work on stay/switch decisions has proposed they originate from frontopolar cortex [49]. Hence, processes in the RL unit might be best explained by a neural circuit between brainstem, aMFC and frontopolar cortex.

The Control unit was adopted from [30]. Its first part contains units that point to specific posterior processing areas, indicating which neurons should be (un)bound. Thus, this area stores the task demands. We labeled this part LFC, given the prominent role of LFC in this regard [50], [51]. The second part of the Control unit sends random bursts to posterior processing areas to synchronize currently active areas. Given the prominent anatomical connectivity of pMFC to motor control and several posterior processing areas [48], we tentatively label this part pMFC. The efficiency of this controlling process is largely determined by pMFC theta power: More power leads to more and longer bursts [30]. This is consistent with empirical work linking high MFC theta power to efficient cognitive control [34], [35]. Power in the model pMFC is itself modulated by the occurrence of negative prediction errors. More specifically, when a negative prediction error occurs, the pMFC node will receive bursts, which will increase pMFC theta power. In absence of negative prediction errors, this theta power will slowly decrease across trials. This is consistent with the idea that a constant high MFC power might be computationally suboptimal and empirically implausible. For instance, MFC projects to locus coeruleus (LC;[52]); LC firing is thought to be cognitively costly, perhaps because it leads to waste product in the cortex that needs to be disposed [36]. In sum, in the Control unit, LFC and pMFC jointly align neural synchronization in modules of the Processing unit to meet current task demands [53], [54]. The LFC indicates which modules should be (de)synchronized, and the pMFC exerts control over the oscillations in the Processing unit by (de)synchronizing them via random bursts.

Crucially, both parts of the Control unit use prediction errors, but at a different time scale. More specifically, the pMFC uses an evaluation of the last prediction error to evaluate the amount of control that should be exerted (fast time scale). Hence, when an error occurs, the model will initially exert more control on the currently used task module/strategy. The LFC on the other hand, is guided by processes in the Switch neuron of the RL unit which evaluates prediction errors on a slow time scale by integrating them over multiple trials, in order to decide between staying with the current task module or switching to another. Therefore, if negative prediction errors keep on occurring after the model increased control, it will switch modules/strategies.

### Experimental predictions

Importantly, our model makes several predictions for empirical data. First, it predicts significant changes in the phase coupling between different posterior neo-cortical brain areas after a task switch. Here, we suggest that desynchronization may be important to disengage from the current task. Consistently, [55] found that strong desynchronization marked the period from the moment of disambiguation of ambiguous stimuli to motor responses. Additionally, Parkinson disease patients, often characterized by extreme cognitive rigidity, show abnormally synchronized oscillatory activity [56]. Thus, we suggest that neural synchronization between task-relevant brain areas is crucial for implementing task rules. Additionally, desynchronization is necessary for disengaging from a task.

Second, we explored midfrontal theta-activation in the time-frequency domain by wavelet convolution. These analyses showed an increase of theta power after an error. This was caused by bursts that were sent from the RL unit as described in Eq (8). Hence, the model predicts an increase of theta amplitude in the MFC after negative prediction errors in tasks where these prediction errors signal the need for increased cognitive control [34], [35], [37].

Third, we connected the model to research demonstrating theta/gamma interactions where faster gamma frequencies, which implement bottom-up processes, are typically embedded in, and modulated by, slower theta-oscillations, in order to implement top-down processes [40], [57–59]. For this purpose, we considered coupling between pMFC theta phase and gamma amplitude in the Processing unit. Our model predicts a strong PAC increase in the first trial(s) after a task switch, which decays slowly after the switch. This reflects the binding by random bursts control process which is increased after task switches, and decays once a task rule is sufficiently implemented. Hence, the model predicts a strong coupling between frontal theta phase and posterior gamma amplitude when new task rules need to be implemented.

### Limitations and extensions

The model contained several limitations, and consequently also possibilities for future extensions. First, the RL unit currently learns to assign a value to some task module. It can determine when a task switch occurred, and then make a binary switch assessment; to switch or not to switch to another task module. Thus, when the model realizes that the current task module/strategy is incompatible with the current task/environment, it has to change its behavior. It will attempt random strategies until an appropriate one is found. Learning *when* to switch can be considered as a type of meta-learning. However, the full model would benefit significantly from more advanced meta-learning mechanisms. Future work will address this issue by adding second level (contextual) features which allow the LFC to (learn to) infer *which* of multiple task modules should be synchronized. One useful application of such second level features would be task set clustering, which allows to generalize quickly over multiple contexts. Specifically, if a novel second-level feature becomes connected to an earlier learned task set (in LFC), all the task-specific mappings of this task set would immediately generalize to the novel second-level feature. This is consistent with immediate generalization seen in humans [60–62].

Second, several parameters of the model were fixed, but might more generally be controllable (learnable) as well. For example, the time scale of the Switch neuron is controllable by the *σ* parameter in Eq (12). In a very stable environment, a low *σ* is adaptive, which slows down the time scale, decreasing the weight of more recent prediction errors. Instead, if the environment is unstable, a less conservative strategy is in order (high *σ*), in which case the model accumulates evidence across less trials in order to make a switch decision. Earlier models already described how switching between hypotheses could depend on environmental stability and noise [63]; such manipulation (here, of parameter *σ*) might be usefully implemented in future developments of the current model too.

Third, although using negative prediction errors to modulate the control amplitude of the pMFC is efficient in the current context, this might not be ideal in more complex environments. Thus, another future challenge is broadening the control signal (i.e., beyond negative prediction errors) that the model uses to optimally adapt to the environment’s reward and cost structure [45].

Fourth, the node architecture of neuronal triplets is an oversimplification of how oscillations are produced in the human brain. Several neural models propose that interacting excitatory (E) cells and inhibitory (I) cells generate oscillations [33], [64]. These oscillatory neurons are grouped with stimulus-driven neurons in cortical columns; oscillatory neurons modulate the activation of the stimulus-driven neurons [65]. In the current model, these assumptions are implemented in the simplest way, namely where each column consists of just three neurons (*E*, *I*, and *x*), and the oscillatory activity modulates the stimulus-driven activity. Furthermore, our implementation of processing within a neuronal triplet is perhaps biologically implausible, in the sense that the neuron that processes stimuli (*x*) is distinct from the neurons that generate the oscillations (*E*, *I*) which do not process any stimulus information. Future work will determine whether the current approach can be scaled to more biologically plausible architectures.

Fifth, the model ignored some aspects of oscillatory dynamics. For instance, our model only implements neural synchronization between Processing unit neurons with the same (gamma-band) frequencies. This scenario might be unrealistic in a typically noisy human brain. However, the problem of noise can be efficiently solved by employing rhythmic bursts, such as the theta-frequency we implemented here. Specifically, one-shot synchronizing bursts would cause oscillations with (slightly) different (gamma-band) frequencies to gradually drift apart after the burst. With rhythmically paced bursts, the gamma oscillations have no time to drift apart since the next burst occurs before the drift becomes substantial. In line with this idea, previous work has demonstrated how the model can deal with gamma frequency differences of at least around 2% [30]. Moreover, one might wonder if it would be optimal to send bursts at a frequency much faster than theta, thus providing no opportunity for noisy oscillations to drift apart. However, the current work showed that accuracy of the model dramatically declines if the pMFC sends bursts at a faster frequency than theta. The reason is that bursts given by the pMFC to the Processing unit introduce noise to the system. This can be clearly observed in Fig 3C, in which there are short periods of irrelevant neuronal activation during the bursts. Hence, an optimal agent would want to limit the bursts as much as possible. Since these bursts are phase locked to the pMFC oscillation and rely on its amplitude, the model performs best with slower pMFC frequencies that are rapidly attracted (high *Damp*) towards a small amplitude (low *r*_*min*_). Again, the oscillations in the Processing unit of the current model all have the exact same frequency. When Processing unit activations do not have the same frequency, we thus conjecture that there is an optimal, intermediate (theta) bursting frequency, depending on the Processing unit (gamma) frequency. Future work should explore such an optimal balance between a Controller/ bursting (theta) frequency and a Processing (gamma) frequency in more noisy systems. Another aspect of oscillatory dynamics we ignored is that BBS may be more biologically plausible, and more efficient, with small inter-areal delays [66]. Future work will consider an additional (meta-) learning mechanism that learns to synchronize nodes with an optimal phase delay between task modules.

### Related work

The current work relies heavily on previous modeling work of cognitive control processes. For instance, in the current model the LFC functions as a holder of task sets which bias lower-level processing pathways [29], [67]. It does this in cooperation with the MFC. Here, the aMFC determines when to switch between lower-level task modules. Additionally, also the amount of control/ effort that is exerted in the model is determined by the RL processes in the aMFC[44–46]. More specifically, negative prediction errors will determine the amount of control that is needed by strongly increasing the pMFC signal [42]. This is consistent with earlier work proposing a key role of MFC in effort allocation [44], [45], [68].

In the current model, the MFC, together with the LFC, functions as a hierarchically higher network that uses RL to estimate its own task-solving proficiency. Based on its estimate of the value of a module, and the reward that accumulates across trials, it evaluates whether the current task strategy is suited for the current environment. Based on this evaluation, it will decide to stay with the current strategy or switch to another. More specifically, the value learned by the RL unit acts as measure of confidence that the model has in its own accuracy. The model uses this measure of confidence to adjust future behavior, a process that has been labeled as meta-cognition [69], [70].This is in line with previous modeling work that described the prefrontal cortex as a reinforcement meta-learner [43], [46–48].

One problem we addressed in this work was the stability-plasticity dilemma. As we described before, previous work on this dilemma can broadly be divided in two classes of solutions. The first class is based on mixing old and new information [2–5]. The second class is based on protection of old information. Our solution also exploited the principle of protection. Future work must develop biologically plausible implementations of the mixing principle too, and investigate to what extent mixing and protection scale up to larger problems.

### Summary

We provided a computationally efficient and empirically testable framework on how the primate brain can address the tradeoff between being sufficiently adaptive to novel information, while retaining valuable earlier regularities (stability-plasticity dilemma). We demonstrated how this problem can be solved by adding fast BBS and RL on top of a classic slow synaptic learning network. RL is used to synchronize task-relevant and desynchronize task-irrelevant modules. This allows high plasticity in task-relevant modules while retaining stability in task-irrelevant modules. Furthermore, we connected the model with empirical findings and provided predictions for future empirical work.

## Methods

### The models

As mentioned before and is shown in Fig 1A, our model consists of three units. First, the Processing unit includes the task-related neural network, which is trained with a classical learning rule (RW, BP or RBM). On top of this classical network, an extra hierarchical layer is added consisting of two units [28]. The RL unit, adopted from the RVPM [24], evaluates whether the Processing unit is synchronizing the correct task modules. This evaluation is used by the Control unit [30] to drive neural synchronization in the Processing unit. Thus, this hierarchically higher network allows the models to implement BBS in an unsupervised manner.

#### The processing unit

An important feature of the current model is that all nodes in the Processing unit consist of triplets of neurons (Fig 2B), as in [30]. The mechanisms of these nodes are illustrated in the Results section and described by Eqs (1)–(8). Importantly, all weights (*W*) in the Processing unit are subject to learning. Here, learning is done according to one of the three classic learning rules; RW, BP or RBM [25–27]. A new learning step was executed at the end of every trial. Because activation in the rate code neurons is modulated by *G*(*E*_*i*_) (see Eq (5)), the activation patterns *x*_*i*_ also oscillate (see Fig 3C). For simplicity, we use their maximum activation across one trial as input for the learning rules, *X*_*i*_ = max(*x*_*i*_). Importantly, the standard formulation of the Rescorla-Wagner rule does not combine well with the full model because, in this combination also non-active units would be able learn. To remedy this, a small adjustment was made to the learning rule [25] for the full model. Specifically, we added one term to the classic rule in order to only make co-activated neurons eligible for learning, resulting in

$$\Delta W_{io}=\beta \times (Target-X_{o})\times X_{i}\times X_{o}$$ (9)

in which β is the learning rate parameter. Importantly, this adjustment of the learning rule also results in a plasticity cost. More specifically, plasticity decreases because the added term (*X*_*O*_) represents the activation of the output unit, which is typically lower than 1 and hence slows down learning. Because the no-synchrony model obtains no advantage of this adjusted learning rule and we aimed to give the classic model the best chances for competing with the full model, we only used the adjusted learning rule (Eq (9)) for the full model.

For the RW and BP networks, a trial ended after 500 time steps (1 sec). Here, the first 250 time steps (500 msec) were simulated as an inter-trial interval in which the Rate code neurons (*x*) did not receive input. In the next 250 time steps, input was presented to the networks. The RBM network also started a trial with 250 time steps without stimulation of the Rate code neurons. After this inter-trial interval the network employs iterations of bidirectional information flow to estimate the necessary synaptic change [27]. We used 5 iterations. Every iteration step (2 in one iteration; one step for each direction of information flow) lasted for 250 time steps. The RBM algorithm also employs stochastic binarization of activation levels at each iteration step. Also here, we used the maximum activation over all time steps (*X*_*i*_) to extract a binary input for that neuron in the next iteration step.

As mentioned in the main text, we compare our new (full) models to models that only use synaptic learning and hence do not use synchronization (no-synchrony models). Thus, those no-synchrony models only have a Processing unit. Here, all used equations and parameters are the same as described above, except for the no-synchrony RW model where we use the classic learning rule instead of the one described in Eq (9). The only difference is that they do not have phase code neurons and by consequence, *G*(*E*_*i*_(*t*)) = 1 in Eq (5).

#### The RL unit

As RL unit, we implemented the Reward Value Prediction Model (RVPM; 9). Here, there is one expected reward neuron, *V*, which holds an estimation of the reward the model will receive given the task module it used. This estimation is made by

$$V=Z^{T}\times (LFC+1)/2$$ (10)

In this equation, ***Z*** is a (column) vector representing the synaptic connections from LFC neurons to the *V* neuron as presented in Fig 2A. This vector holds information about the value of specific task modules. Superscript *T* indicates that we transposed the ***Z*** vector. The ***LFC***-term is a vector of LFC values representing which task module drove network behavior on the current trial. These values are normalized, controlling for the fact that LFC neurons can take on negative values. Hence, *V* will represent the expected value of the task module that is synchronized by the LFC represented in the ***Z*** vector. These weights are updated by the RVPM learning rule [24],

$$\Delta Q_{i}={\alpha \times V_{n}\times (LFC}_{i}+1)/2\times (\delta^{+}-\delta^{-})$$ (11)

which is a reinforcement-modulated Hebbian learning rule from the broader class of RL algorithms. Here, *n* represents the current trial. The learning rate, *α*, is set to .01 for the BP and RBM models and to .1 for the RW model. All neurons in the RL unit, are rate code neurons which have no time index because they only take one value per trial.

Two prediction error neurons in the RL unit compare the estimated reward (*V*) with the actual received reward. This leads to a negative prediction error *δ—*> 0 if the reward is smaller than predicted, *δ* ^**+**^ > 0 if the reward is larger than predicted, and *δ— = δ* ^***+***^ = 0 if the prediction matches the actual reward (see [24] for more details). In the current model, the Switch neuron will accumulate this prediction error signal in order to evaluate whether the task rule has changed or not. For this purpose, it follows,

$$S_{n+1}=\sigma \times S_{n}+(1-\sigma )\times \delta_{n}^{-}$$ (12)

Here, the value of *σ* is set to .8 for the multi-layer models and .5 for the RW model. When activation in this neuron reaches a threshold of .5, it signals the need for a switch to the LFC in the Control unit and resets its own activation to zero. In the equation, *n* refers to the trial number. Hence, a sequence of big negative prediction errors will cause activation in the Switch neuron to reach the threshold.

#### The control unit

*A*s in previous work [30], the Control unit consists of two parts, corresponding to posterior medial (pMFC) and lateral (LFC) parts of the primate prefrontal cortex.

The modelled pMFC represents one node (Fig 2) consisting of one phase code pair (*E*_pMFC_, *I*_pMFC_) and a rate code neuron (*pMFC*). The specifics of this pMFC node are described in the Results section and illustrated in Fig 3A.

In general, the model implements a “win stay, lose shift” strategy, shifting attention in LFC when reward appears less than expected. As shown in Fig 2A, the LFC consists of four rate code neurons that each have a pointer to one (or two) of the different modules in the Processing unit. Three of these LFC neurons are each connected to one of the three task modules in layer 2. For these LFC neurons, at trial *n* = 1 a random choice is made where one neuron is set to 1 and the others to -1. These activations remain until activation in the Switch neuron, S, reaches the threshold. At this point, a new choice is made where one LFC neuron is set to 1 and all others are set to -1. This choice is based on a softmax decision rule,

$${Selection\;probability}_{i}=\frac{e^{\left(Q_{i}+{Inh}_{i}\right)}}{\sum_{j}e^{\left(Q_{j}+{Inh}_{j}\right)}}$$ (13)

Here, *Q*_*i*_ is the value associated with that LFC neuron/ task module and *Inh*_*i*_ is an inhibition signal. At the moment the switch threshold is reached, *Inh*_*i*_ is set to -2 for the currently synchronized module. This value decays by 10% on every trial afterwards. This inhibition is implemented to avoid that the model would constantly switch between two modules. Because non-chosen LFC neurons are set to -1, the network always synchronizes one task module with layer 1 for the RW model and with layers 1 and 3 for the BP and RBM models, and desynchronizes the other task modules. When it realizes that the task rule has switched, it will select a new task module. For this selection it will prioritize task modules that have a high value assigned to it (encoded in *Q*_*i*_), except when this task module was presented recently; in that case, the task module is inhibited (*Inh*_*i*_). The remaining LFC neuron is connected (constant value of 1) to the other layers (1 and 3) of the network that must be synchronized.

### The task

We test our model on a reversal learning task [71], [72]. We divide the task in six equally long task blocks. In the first three blocks, the model should learn three different new task rules (rule A, B and C in blocks 1, 2 and 3 respectively). In the second half, the model has to switch back to the previously learned rules (rule A, B and C in blocks 4, 5 and 6 respectively).

For the RW network, we use a one-dimensional task. This task consisted of 360 trials. Here, on each trial one out of three stimulus features is activated. For every task rule we link a stimulus-feature to a response option. More specifically, in task rule A, feature 1 (F1) is associated to response (R1), feature 2 (F2) to response 2 (R2) and feature 3 (F3) response 3 (R3). In task rule B, F1 is associated to R2, F2 to R3 and F3 to R1. Task rule C associates F1 to R3, F2 to R1 and F3 to R2. All stimuli are presented equally often in random order.

For the BP and RBM networks, a multi-dimensional task is used consisting of 3600 trials. In order to gain insight in how the complexity of the task affects our model, we implemented a task with two stimulus dimensions (two-dimensional task) and one with three stimulus dimensions (three-dimensional task). For the RBM model, we only implemented the three-dimensional task. Every stimulus dimension has three features. In total, a task consists of *N* + 1 dimensions, in which *N* is the number of stimulus dimensions and the extra dimension is a cue dimension (with *N* features), indicating which of the *N* stimulus dimensions is relevant on the current trial. On each trial one feature of every dimension is activated. In line with the one-dimensional task, the *N* = 3 task features of the stimulus dimensions are, within each task rule, linked to one response option. Again, in each block, each possible stimulus is presented equally often, in a random order.

### Simulations

To test the generality of our findings, we varied the synaptic learning rate. This parameter was varied from 0 to 1 in 11 steps of .1. For each value, we performed 10 replications of the simulation. In every simulation, the strength of synaptic connections at trial 1 was a random number drawn from the uniform distribution, multiplied by half the bias value (and 1 for the RW based model).

The effects of other model parameters were already demonstrated in previous work [24], [30], but we again validated that the model shows qualitatively similar patterns when we varied some of the parameters. A table of all parameter values used in both the original simulations and parameter explorations is provided in the S1 Text. Specifically, we explored different frequencies (*C* in Eqs (1) and (2)) in the Processing unit and the pMFC. Additionally, we also explored the *Damp* and *r*_*min*_ parameters in the pMFC (again Eqs (1) and (2)). For this simulation we used the RW model with β = .2. We fully crossed all parameter values for *C*, *Damp* and *r*_*min*._ and performed 5 replications. In a separate set of explorations, we varied *σ* and *α* in the RL unit (see Eqs (11) and (12)) for both the RW and BP algorithm, for both a slow and a fast-synaptic learning rate (β). Again, we performed 5 replications for each parameter combination. Results of the latter parameter exploration are described in the S1 Text.

### Statistical analyses

For the purpose of comparison, we divided the trials of the task for every model into 120 bins. For the RW model, bin size equals 3 trials; for the BP and RBM models, bin size equals 30 trials. We evaluate the performance of our model on several levels. First, we evaluate overall task accuracy. Second, we evaluate plasticity. For this purpose, we explore the performance of the model during the first 5 bins of the first 3 blocks. Hence, we test how quickly a model can learn a new task rule. Third, we evaluate stability. In particular, we explore the interference of learning other task rules in between two periods of performing the same task rule. For this purpose, we compare the accuracy during the first 5 trial bins of block 4, 5 and 6 with the last 5 trial bins of block 1, 2 and 3. If the model saved what was learned, this difference should be zero. If the model displays catastrophic forgetting, it would have a negative stability score.

Importantly, we also connect with empirical data and describe testable hypotheses for future empirical work. As a measure of phase synchronization between excitatory neurons in the Processing unit, we compute the correlation at phase lag zero. A correlation of 1 indicates complete synchronization and -1 indicates complete desynchronization. Phase-amplitude coupling (PAC) is computed as the debiased phase-amplitude coupling measure (dPAC; [73]) in each trial. Here,

$$dPAC=|\frac{1}{h}\sum_{t=1}^{h}a_{t}{\times (e}^{i\varphi_{t}}-\Phi^{-})|$$ (14)

in which

$$\Phi^{-}=\frac{1}{h}\sum_{t=1}^{h}e^{i\varphi t}$$ (15)

In these equations, *t* represents one time step in a trial, *h* is the number of time steps in a trial, *a* is the amplitude, *φ* is the phase of a signal, and *i*^2^ = -1. In the current paper, we are interested in the coupling between the phase of the theta oscillation in the pMFC node of the Control unit and the gamma amplitude in the Processing unit. Phase was extracted by taking the analytical phase after a Hilbert transform. The gamma amplitude was derived as the mean of the excitatory phase code activation of all nodes in the Processing unit by

$$a_{t}=\frac{1}{I}\sum_{i=1}^{I}\left|E_{it}\right|$$ (16)

with *I* being the number of nodes in the Processing unit, *t* referring to time and *E*_*i*_ being the respective excitatory phase code neuron.

For all measures, we represent the mean value over *Nrep* = 10 replications and error bars or shades show the confidence interval computed by mean± 2×(SD/√*Nrep*).

Additionally, we evaluated the pMFC theta activation. More specifically, time–frequency signal decomposition was performed by convolving the signal of *E*_pMFC_ by complex Morlet wavelets, $e^{i2\pi ft}e^{-t^{2}/(2\sigma^{2})}$, where *i*^2^ = -1, *t* is time, *f* is frequency, ranging from 1 to 10 in 10 linearly spaced steps, and σ = 4/(2πf) is the “width” of the wavelet. Power at time step *t* was then computed as the squared magnitude of the complex signal at time t and frequency f. We averaged this power over all simulations and all replications of our simulations. This power was evaluated by taking the contrast between the inter-trial intervals following correct (1) and error (0) reward feedback.

### Data and software availability

Matlab codes that were used for both the model simulations and data analysis are available on GitHub (https://github.com/CogComNeuroSci/PieterV_public).

## Supporting information

S1 Text Supplementary materials. We present results for the RBM model simulation and exploration of the parameters in the RL unit. Additionally, we provide tables of all parameter values that were used for our simulations.
(DOCX)

S1 Fig The RBM model. The first row (A-C) gives a deeper insight into the model dynamics. In A, orange lines represent the synaptic model and blue lines the full model. In E, brown lines represent the first chosen task module, magenta lines the secondly chosen module and green lines the remaining task module. In F, the horizontal blue line indicates the Switch threshold and the yellow arrows mark the moment the activation reached the threshold. The second row (D-F), shows the mean accuracy, plasticity and stability for the RBM model across learning rates. Again, orange represents the synaptic model and blue the full model Overall, red vertical dashed lines indicate task switches, black horizontal dashed lines indicate chance level of accuracy, and shades represent 95% confidence intervals.
(TIF)

S2 Fig RL unit parameter exploration. Mean accuracy is shown for all simulations with a certain parameter value. The first row (A-D) shows results for the RW model and the second row (E-H) for the BP model. The first two columns (A, B, E, F) show data for simulations with a small synaptic learning rate (β = .2) for different values of *α* and *σ* respectively. The last two columns (C, D, G, H) show the same data for a faster synaptic learning rate (β = .8). Black vertical dashed lines indicate the parameter values that were used for the original simulations described in the main text.
(TIF)
