# Supplementary material for: Learning to synchronize: How biological agents can couple neural task modules for dealing with the stability-plasticity dilemma
Source: PLoS Comput Biol. 2019 Aug 20;15(8):e1006604. doi: 10.1371/journal.pcbi.1006604 (PMC6716678; doi:10.1371/journal.pcbi.1006604)
Supplement: S1 Text — We present results for the RBM model simulation and exploration of the parameters in the RL unit. Additionally, we provide tables of all parameter values that were used for our simulations. (DOCX) [file pcbi.1006604.s001.docx]

**Supplementary materials**

**RBM**

Figure S1 shows the results for model simulation on the three-dimensional task with the RBM algorithm. The general model mechanisms, such as the Switching neuron and the synchronization, are very similar to results with the BP and RW models shown in the main text. Additionally, also with the RBM algorithm, our full model provides a significant improvement in terms of overall accuracy relative to the synaptic model. In contrast to the BP model however, the synaptic RBM model shows no disadvantage over the full model in terms of plasticity. Since the RBM algorithm learns much faster than BP, the stability problem decreases for higher learning rates. Nevertheless, even at these higher learning rates, the full model has a significant advantage in stability, so also with RBM, the full model’s overall performance is better.

**Figure S1.** *The RBM model.* The first row (A-C) gives a deeper insight into the model dynamics. In S1A, orange lines represent the synaptic model and blue lines the full model. In E, brown lines represent the first chosen task module, magenta lines the secondly chosen module and green lines the remaining task module. In F, the horizontal blue line indicates the Switch threshold and the yellow arrows mark the moment the activation reached the threshold. The second row (D-F), shows the mean accuracy, plasticity and stability for the RBM model across learning rates. Again, orange represents the synaptic model and blue the full model Overall, red vertical dashed lines indicate task switches, black horizontal dashed lines indicate chance level of accuracy, and shades represent 95% confidence intervals.

**Parameter exploration for the RL unit**

In the main text, we described parameter exploration of the Processing unit. Here, we report additional simulations in which we explored several parameter values for the RL unit of the model (see Table B). We did this for the BP and RW model and for a slow (β = .2; Fig. S2A, B, E, F) and a fast (β = .8; Fig. S2C, D, G, H) synaptic learning rate. We performed 5 replications of every possible parameter combination. More specifically, we varied the RL unit learning rate (α) and prediction error buildup rate (σ) parameters of the RL unit in equations (11) and (12) respectively.

For the RW model (first row of Fig S2), we observed that the model performs best with a small-to-intermediate α (Fig. S2A). Hence, in the current task, the model performs optimally when it learns the value of a task module (*V*) rather slowly. This is because if the estimation of *V* fluctuates too much, the prediction errors are not stable enough to make an accurate evaluation of how well the model is performing. Additionally, the RW model obtains a better accuracy with a small σ (Fig. S2B). Thus, the RW model should assign a large weight (1-σ) to spontaneous prediction errors (*δ ^-^*). This follows from the fact that the RW algorithm is very efficient and makes almost no errors. Also, note that the task provides deterministic feedback. Hence, if one is completely certain of one’s response but did not receive reward, one can be certain that the task rule has changed. In this case, one error is enough to make a switch decision. A similar pattern is observed for both the slow (β = .2) and fast (β = .8) synaptic learning rate.

For the BP model (second row of Fig. S2), we observed that the model performs optimally with a very small α. Again, this is necessary to obtain a stable estimate of the prediction errors. In contrast to the RW model, the BP model performs best with a very small σ (see Fig S2E, G). This is a consequence of the fact that the BP algorithm learns much slower and therefore makes more mistakes. Hence, it should be more conservative and not switch task modules after one error. Again, we observe a similar pattern for the slow (β = .2) and fast (β = .8) synaptic learning rate.

**Figure S2.** *RL unit parameter exploration.* Mean accuracy is shown for all simulations with a certain parameter value. The first row (A-D) shows results for the RW model and the second row (E-H) for the BP model. The first two columns (A, B, E, F) show data for simulations with a small synaptic learning rate (β = .2) for different values of *α* and *σ* respectively. The last two columns (C, D, G, H) show the same data for a faster synaptic learning rate (β = .8). Black vertical dashed lines indicate the parameter values that were used for the original simulations described in the main text.

**Supplementary tables**

In Table A, we present the parameter values that were used for the original simulations (main text and RBM). Since we always used the same parameters for the BP and RBM models, we grouped these as multi-layer models. We also shortly describe the function of each parameter. Parameters that were explored in extra simulations are indicated by stars in Table A. Parameters that were explored in the main text (Processing and Control units), are indicated with one star (*); parameters that were explored in the Supplementary Materials (RL unit) are indicated with two stars (**). The exact parameter values for these explorations are given in Table B.

| Algorithms | **RW** | **Multi-layer models**  **(BP and RBM)** | |  | |
| --- | --- | --- | --- | --- | --- |
| Parameters | Parameter values | | Function | |  |
| Processing unit |  |  |  | |  |
| *C** | .58 | .58 | Determines frequency of Input processing | |  |
| *Damp* | .3 | .3 | Attraction of amplitude to baseline level | |  |
| *r*_min_ | 1 | 1 | Baseline level of amplitude | |  |
| bias | 0 | 5 | Reach near-zero activation levels in absence of input | |  |
| β**** | 0 to 1 in steps of .1 | 0 to 1 in steps of .1 | Synaptic learning rate | |  |
| Control unit |  |  |  | |  |
| *C** | .07 | .07 | Determines  Controller frequency | |  |
| *Damp** | .003 | .003 | Slow decay of amplitude | |  |
| *r_min_** | .05 | .05 | Keep baseline activation low | |  |
| RL unit |  |  |  | |  |
| *σ*** | .5 | .8 | Weight given to a single error | |  |
| Switch Threshold | .5 | .5 | Threshold for switching between modules | |  |
| *α*** | .1 | .01 | Learning rate of *V* | |  |

**Table A.** *Parameters used for the original simulations of all three learning algorithms.* Parameters that were explored in additional simulations are indicated with stars (*).

| Simulation | **Main text exploration (RW)** | **Supplementary Material exploration**  **(RW and BP)** |  |
| --- | --- | --- | --- |
| Parameters | Parameter values | |  |
| Processing unit |  |  |  |
| *C** | .15 to .65 in steps of .1 | .58 |  |
| *β*** | .2 | [.2; .8] |  |
| Control unit |  |  |  |
| *C** | .03 to .15 in steps of .2 | .07 |  |
| *Damp** | [0; .003; .03; .3] | .003 |  |
| *r_min_** | [0; .05; .5; 1] | .05 |  |
| RL unit |  |  |  |
| *σ*** | .5 | [.2; .5; .8] |  |
| $\alpha$**** | .1 | [.01; .05; .1; .2] |  |

**Table B.** *Parameters used for explorative simulations.* Unreported parameters adopt the values from the original simulation as presented in Table A. The *C* parameters in both the Processing and control unit were varied in specific steps, other parameters were explored at the values in the square brackets. Parameters indicated with one star (*) are explored in the main text; parameters indicated with two stars (**) are explored in the Supplementary Material exploration.
