# Supplementary figures and images for: Learning to synchronize: How biological agents can couple neural task modules for dealing with the stability-plasticity dilemma
Source: PLoS Comput Biol. 2019 Aug 20;15(8):e1006604. doi: 10.1371/journal.pcbi.1006604 (PMC6716678; doi:10.1371/journal.pcbi.1006604)

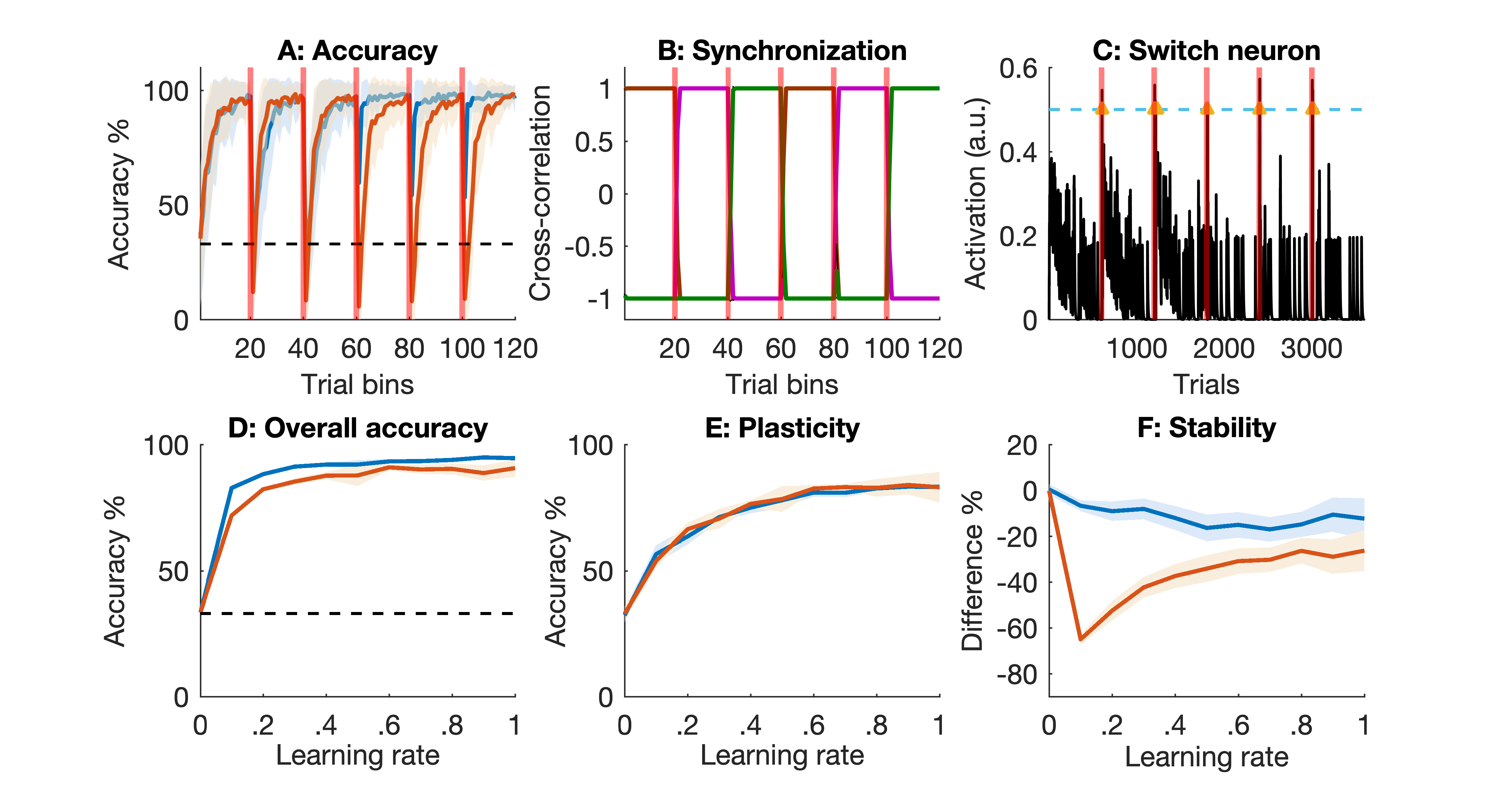

Supplement: S1 Fig — The first row (A-C) gives a deeper insight into the model dynamics. In A, orange lines represent the synaptic model and blue lines the full model. In E, brown lines represent the first chosen task module, magenta lines the secondly chosen module and green lines the remaining task module. In F, the horizontal blue line indicates the Switch threshold and the yellow arrows mark the moment the activation reached the threshold. The second row (D-F), shows the mean accuracy, plasticity and stability for the RBM model across learning rates. Again, orange represents the synaptic model and blue the full model Overall, red vertical dashed lines indicate task switches, black horizontal dashed lines indicate chance level of accuracy, and shades represent 95% confidence intervals. (TIF) [file pcbi.1006604.s002.tif]

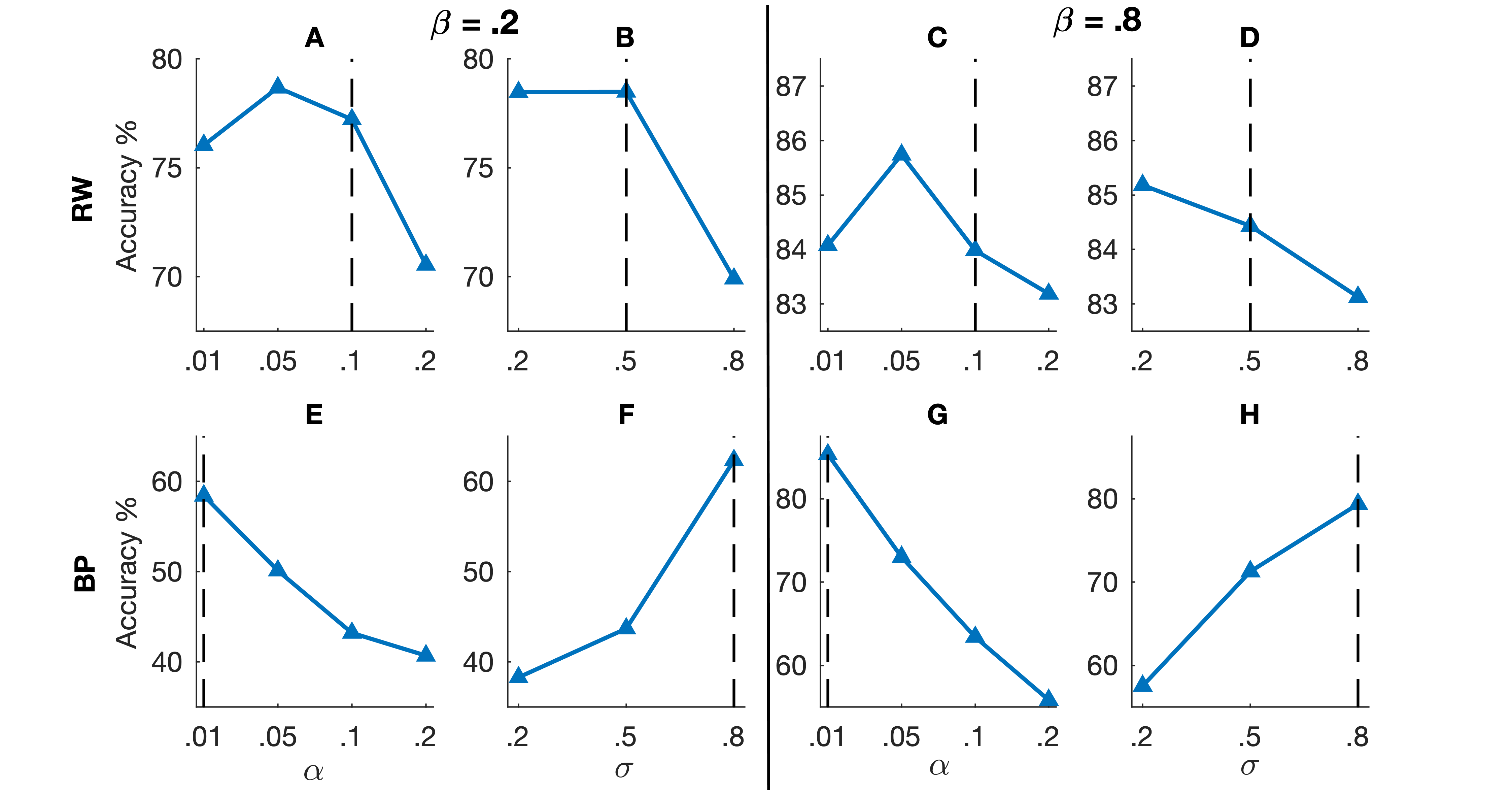

Supplement: S2 Fig — Mean accuracy is shown for all simulations with a certain parameter value. The first row (A-D) shows results for the RW model and the second row (E-H) for the BP model. The first two columns (A, B, E, F) show data for simulations with a small synaptic learning rate (β = .2) for different values of α and σ respectively. The last two columns (C, D, G, H) show the same data for a faster synaptic learning rate (β = .8). Black vertical dashed lines indicate the parameter values that were used for the original simulations described in the main text. (TIF) [file pcbi.1006604.s003.tif]
